# Domain-Specific Cognitive Prosthesis for Face Memory and Recognition

**DOI:** 10.3390/diagnostics12092242

**Published:** 2022-09-16

**Authors:** Jowy Tani, Yao-Hua Yang, Chao-Min Chen, Co Yih Siow, Tsui-San Chang, Kai Yang, Jack Yao, Chaur-Jong Hu, Jia-Ying Sung

**Affiliations:** 1 Department of Neurology, Wan Fang Hospital, Taipei Medical University, Taipei 116079, Taiwan; 2 Biomed Innovation Center, Wan Fang Hospital, Taipei Medical University, Taipei 116079, Taiwan; 3 Department of Neurology, School of Medicine, College of Medicine, Taipei Medical University, Taipei 110301, Taiwan; 4 Taipei Medical University Biomed Accelerator, Taipei Medical University, Taipei 106339, Taiwan; 5 Taipei Medical University Biodesign Center, Taipei Medical University, Taipei 106339, Taiwan; 6 Taipei Neuroscience Institute, Taipei Medical University, New Taipei City 235041, Taiwan; 7 Department of Physical Medicine and Rehabilitation, Taipei Medical University Hospital, Taipei 110301, Taiwan; 8 MediXgraph Inc., Fremont, CA 94555, USA; 9 Department of Neurology, Shuang Ho Hospital, Taipei Medical University, New Taipei City 235041, Taiwan

**Keywords:** cognitive prosthesis, cognitive impairment, face recognition, functional MRI, facial memory, machine learning

## Abstract

The present study proposes a cognitive prosthesis device for face memory impairment as a proof-of-concept for the domain-specific cognitive prosthesis. Healthy subjects (*n* = 6) and a patient with poor face memory were enrolled. An acquaintance face recognition test with and without the use of cognitive prosthesis for face memory impairment, face recognition tests, quality of life, neuropsychological assessments, and machine learning performance of the cognitive prosthesis were followed-up throughout four weeks of real-world device use by the patient. The healthy subjects had an accuracy of 92.38 ± 4.41% and reaction time of 1.27 ± 0.12 s in the initial attempt of the acquaintance face recognition test, which changed to 80.48 ± 6.23% (*p* = 0.06) and 2.11 ± 0.20 s (*p* < 0.01) with prosthesis use. The patient had an accuracy of 74.29% and a reaction time of 6.65 s, which improved to 94.29% and 3.28 s with prosthesis use. After four weeks, the patient’s unassisted accuracy and reaction time improved to 100% and 1.23 s. Functional MRI study revealed activation of the left superior temporal lobe during face recognition task without prosthesis use and activation of the right precentral motor area with prosthesis use. The prosthesis could improve the patient’s performance by bypassing the brain area inefficient for facial recognition and employing the area more efficiently for the cognitive task.

## 1. Introduction

Cognitive deficits may affect one or more cognitive domains [1]. Common causes of cognitive deficits include degenerative processes [2], stroke [3], and traumatic brain injury [4]. Various acquired brain insults could also lead to cognitive deficits in one or more domains [5].

Patients with cognitive deficits suffer from significant deterioration of quality of life [6,7]. The condition also inflicts a heavy burden of care on families and society [8]. The global cost of dementia alone was estimated at USD 1 trillion annually [9].

Common domain dysfunction seen in patients with cognitive impairments includes memory impairment, impairment of executive function, visuospatial dysfunction, and impairment in language function. Patients with cognitive impairments could have a unique combination of the above dysfunctions in one or more domains. The medical devices aimed at dementia and other cognitive dysfunction currently focus on the nonspecific enhancement of cognitive function instead of alleviating domain-specific cognitive dysfunction [10]. Nevertheless, nonspecific enhancement of cognitive function may not necessarily provide the optimal support needed to enable the patient’s functionality in daily life; instead, the availability of a cognitive prosthesis system that focuses on individual patients’ domain-specific cognitive dysfunction might serve the patients’ needs better [11]. A domain-specific cognitive prosthesis could be able to provide real-time cognitive assistance to alleviate patients’ specific cognitive deficits and help them through their daily challenges.

Advances in machine learning techniques, edge computing, and wearable technology have made real-time cognitive assistance possible. Various visual- and auditory-based cognitive prosthesis and neural prosthesis have been proposed recently [12,13,14]. Difficulty recognizing acquaintances is one of the issues causing distress in cognitively impaired patients with poor face memory.

Over the years, numerous attempts have been made to develop memory aid strategies using web-based platforms [15,16], mobile devices [16], wearable devices [17,18], or robots [19]. However, there is a lack of studies that investigate the effect of machine-learning-based memory aid on the clinical outcomes of cognitively impaired patients. Meanwhile, machine-learning-based face recognition algorithms have been employed in Kinect-based devices [20], mobile devices [21], and wearable devices [22], mainly for visually impaired patients. Nevertheless, there is a similar lack of published studies discussing how the technology could affect clinical outcomes of patients with impaired face memory. In this article, we propose a real-time cognitive assistance device for patients with poor face memory as a proof-of-concept for a domain-specific cognitive prosthesis.

On the other hand, despite prominent unmet needs, a limited number of medical technology innovations were available for neurological diseases during the past decade. One of the possible reasons is that many neurological disorders have unclear or complex pathophysiology. Nevertheless, advances in functional studies for the nervous system and its increasingly wider availability have allowed innovators to use functional studies in medical technology concept exploration and testing. The functional studies performed at the concept exploration stage could reduce medical technology research and development risk associated with unclear mechanisms of action.

## 2. Materials and Methods

### 2.1. Subjects

Six healthy subjects (all females, mean age 32 ± 3.41 years) and one patient (female, age 29 years) with poor facial memory participated in the present study. The enrollment period for the study was from August 2018 to July 2019. The inclusion criteria for the healthy subjects are: (1) age ≥20 and (2) normal cognition with no specific cognitive deficits; persons with significant acute or chronic illness are excluded. The inclusion criteria for the patients are: (1) age ≥20, (2) poor facial memory as identified by the Taiwan Face Memory Test (TFMT, total score below 2 standard deviation of the age group) [23]. Patients unable to provide informed consent and/or having comorbidities preventing enrollment into the trial (e.g., combined poor visual acuity and poor hearing) were excluded.

### 2.2. Study Framework and Evaluation Criteria

Several frameworks for developing medical devices have been proposed by academic and regulatory institutions [24]. The biodesign innovation process comprises three phases: identify, invent, and implement [25]. In the identify phase, a team identifies numerous unmet clinical needs, selects a top need, and then carefully determines its need criteria. Then, several concepts are generated in the invent phase, and the top concepts are evaluated against the pre-determined need criteria and assessed for intellectual property and regulatory, reimbursement, and business model feasibility. A concept should only be selected for implementation if it is able to meet all the must-have need criteria.

**Figure 1:**
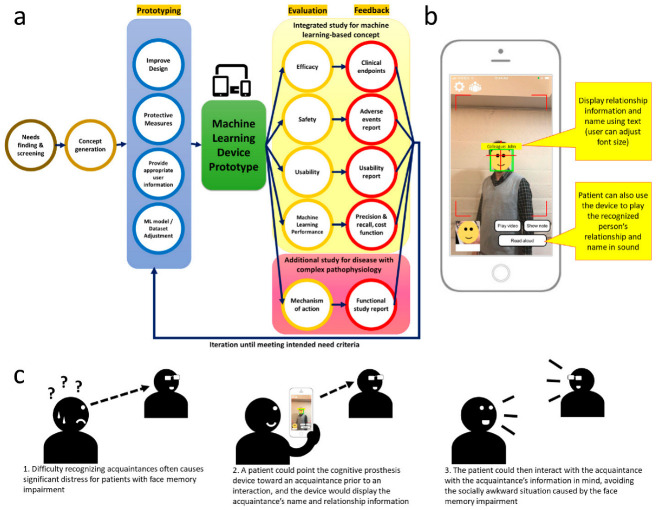
Concept exploration and testing for machine-learning-based concept and disease with complex pathophysiology (**a**), and the cognitive prosthesis for facial recognition device prototype in the present study (**b**). Triptech storyboard for the cognitive prosthesis device is also shown (**c**).

Therefore, concept exploration and testing stood as an important element of the biodesign innovation process. We propose a concept exploration and testing framework for a machine-learning-based medical device in the present study. The framework would employ an integrated study to evaluate the technical and clinical feasibility of a device against a set of criteria adapted from the biodesign need criteria, with minor modifications to account for a machine-learning-based device (Figure 1a).

In the framework, the initial machine-learning-based device prototype is developed based on an unmet need identified through need finding and screening, followed by concept generation and screening. The integrated validation study for the machine-learning-based concept would iteratively evaluate the prototype’s safety, efficacy, usability, and machine learning performance. In assessing the machine learning performance criteria, the framework would determine the precision, recall, and cost function (i.e., the machine learning model’s performance for given data) of the machine learning algorithm. Particularly for diseases with unclear or complex pathophysiology, the functional study could be performed as part of the evaluation to elucidate the disease’s mechanism of action to reduce further research and development risk associated with unclear mechanisms of action.

The evaluation process is tightly associated with device prototyping. Feedback from the evaluation process, including adverse events reports, clinical endpoints, machine learning model performance, usability, and user need profiles, would be used to influence device prototyping. In the prototyping process, the developer would improve the device hardware and software design, add protective measures, provide appropriate user information, and adjust the machine learning model/datasets. The improved device would then undergo another evaluation cycle until it met the requirements.

Need identification through observation and interview with physicians, patients with cognitive impairment, and patient family was conducted in the early phase of the study. Difficulty recognizing acquaintances, i.e., non-familial persons that patients need to interact with daily, was found to be a problem in the patients’ daily lives. The need statement “a way to recognize faces for patients with poor face memory in order to improve recognition of acquaintances” was chosen for further concept exploration and testing, and the following need criteria were then developed based on the above need identification process:

- Efficacy: (must-have) Improved face recognition accuracy and reaction time >50% compared with baseline; (nice-to-have) improved face recognition accuracy and reaction time >75% compared with baseline.
- Safety: (must-have) No serious adverse event.
- Usability: (must-have) Systemic usability scale ≥65; (nice to have) systemic usability scale ≥85.
- Performance: (must-have) Precision ≥90% and recall ≥90% with average training image set <10 images; (nice-to-have) precision ≥95% and recall ≥95% with average training image set <10 images.

### 2.3. The Cognitive Prosthesis Device

The initial concept for cognitive prosthesis for face memory impairment was developed as a possible solution to the need statement. The cognitive prosthesis prototype was based on the Apple iOS platform and operates on the iPhone hardware (Apple Inc., Cupertino, CA, USA). The device was configured to recognize the faces of 7 acquaintances. The machine learning model for face recognition is trained by face photos labeled using information provided by the user. The patient could choose to indicate the facial information through text or voice. The face recognition module of the system employed an open-source platform for machine learning over edge computing devices. It utilized the Apple Core ML machine learning platform for machine learning on the edge computing device. The device used the Apple Vision framework for face detection. It uses facial landmarks such as eyes, mouth, and nose to align the face, and then proceeds to perform face recognition with deep neural network. The machine learning model for face recognition is trained by utilizing pre-trained FaceNet-based [26] Core ML model and customized face images.

The interface of the device is shown in Figure 1b. The device is intended the help a user with poor facial memory to recall the name and relationship information of the acquaintance. The user attempting to recognize the person could point the camera of the device toward the face of that person. The device would automatically attempt to detect any faces captured by the camera and try to recognize the detected faces. If the device can recognize the face, the name and the relationship information of the person will be displayed on the device screen above the face; if the device could not recognize the face, “stranger” would be displayed instead. The user can adjust the font size for the displayed text. The user can also make the device read aloud the person’s name by pressing a button on the screen.

Difficulty recalling acquaintances often causes socially awkward situations for patients with face memory impairment and is a significant source of distress. In daily life, a patient could point the device at an acquaintance prior to an interaction to obtain the acquaintance’s information. The patient would then be able to interact with the acquaintance with the acquaintance’s information in mind. The device’s usage is shown through the triptych storyboard [27] in Figure 1c.

### 2.4. Study Procedures

The healthy subjects would receive the acquaintance face recognition test with and without cognitive prosthesis, as described below. For the patient, before the initial study visit, the study staff provided the patient with the data preparation instructions, which instructed the patient to prepare 6 facial photos of 7 acquaintances that the patient needs to interact with daily but has difficulty recognizing. The photos were used to generate machine learning models for the cognitive prosthesis and for the acquaintance face recognition test. The patient’s clinical, neuropsychological, and quality of life profile were obtained. Neuropsychological tests performed included the Wechsler Adult Intelligence Scale-IV [28], Mini-Mental State Examination (MMSE) [29], Modified Wisconsin Card Sorting Test [30], Wechsler Memory Scale-III [31], visual and spatial perceptual functions [32], Benton’s facial recognition test [33], and TFMT [23].

After the patient completed the initial visit evaluation and received the instructions for device use, the patient received a face recognition assistant device.

The patient was encouraged to use the device to help with the recall of acquaintances in daily life. Efficacy, safety, usability, and machine learning performance are performed at the initial visit and each week thereafter, up to 4 weeks.

### 2.5. Efficacy Evaluation

Several psychometric tests for face recognition capability have been proposed in the literature, and these tests were mainly designed to assess stranger or public figure recognition. To our knowledge, no easily available psychometric test objectively measures a patient’s capability to recognize an acquaintance’s face that they are interacting with daily.

#### 2.5.1. Acquaintance Face Recognition Test

Our team developed a novel “acquaintance face recognition test” using Python (version 3.5, Python Software Foundation) for the present study’s efficacy evaluation. The test was used for the assessment of both healthy subjects and the patients. Moreover, it allows the evaluation of facial recognition performance with or without a cognitive prosthesis device use. Our team also built a version of the test which is compatible with use in conjunction with functional magnetic resonance imaging (fMRI) scanning.

The test comprised a practice section and an acquaintance face recognition testing section. During the testing session, the subjects were instructed to press the “O” or “X” key to indicate whether the face image that appeared on the screen is compatible with the title and name appearing on the screen, as soon as the image and the text appear on the screen. “Accuracy” and “reaction time” of acquaintance face recognition were then measured. Each subject was given two attempts, with 25 face images shown for each of the attempts. The face images in the test appear in random order to minimize the potential training effect.

The healthy subjects and the patient all underwent the same acquaintance face recognition test procedure but with different face images. To assess the subjects’ face recognition performance with cognitive prosthesis use, the subjects were instructed to point the cognitive prosthesis device camera toward the test screen and to use the interpretation result of the device to complete the particular test sections requiring prosthesis use.

#### 2.5.2. Taiwan Face Memory Test, Quality of Life, and Other Neuropsychological Assessments

The TFMT [23], the Short Form Health Survey (SF-36) [34], MMSE, and Benton’s face recognition test were performed during the initial visit and each week afterwards alongside the acquaintance face recognition test for efficacy evaluation.

### 2.6. Safety, Usability, and Machine Learning Performance Evaluation

Safety evaluation was performed at a specified interval and the end of the study. Safety endpoints would be assessed based on adverse event reports. For usability evaluation, task analysis and SUS were performed [35,36].

Machine learning model performance of the multiclass classification face recognition model algorithm was analyzed based on the baseline and real-world weekly device usage data, according to accepted classification tasks performance analysis metrics [37].

### 2.7. Functional Study to Clarify the Mechanism of Action

The fMRI study was performed to elucidate the mechanism of action of the cognitive prosthesis device. A time-course series was acquired with a 3.0-T MRI system. The image data were analyzed using Statistical Parametric Mapping 12 (SPM12; Welcome Department of Imaging Neuroscience, London, UK) implemented in MATLAB (MathWorks, Natick, MA, USA).

The patient was instructed to determine whether the displayed name matched an acquaintance face she recognized or whether the displayed name matched the simulated recognition result of a cognitive prosthesis. The tasks were displayed on a screen and viewed through a prism mirror within an MRI scanner. The task program was controlled by E-prime (Psychology Software Tools, Inc., Pittsburgh, PA, USA).

**Figure 2:**
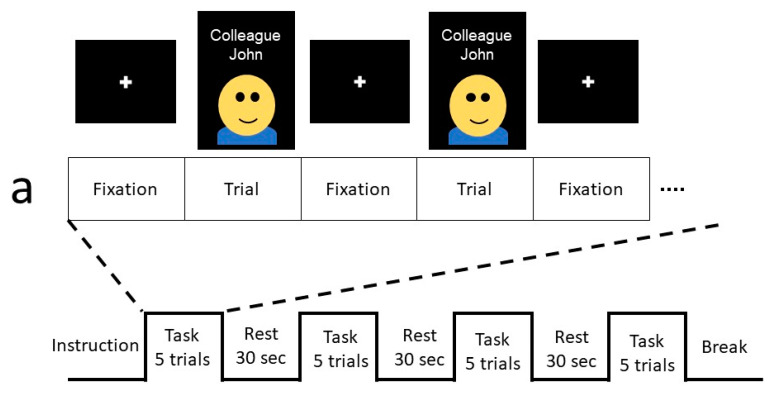

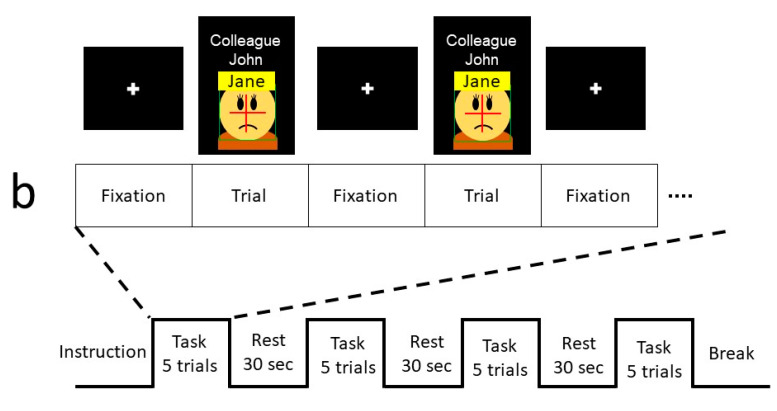
Functional MRI procedure for the present study. Session 1 (**a**) and session 2 (**b**) of the study are shown. The patient was instructed to determine whether the face image matched the name and relationship information displayed on the screen.

The patient received two sessions, as shown in Figure 2. In the first session, the patient was instructed to determine whether the displayed name and relationship information matched an acquaintance face she recognized. In the second session, the patient was instructed to determine whether the displayed name and relationship information matched the simulated recognition result of the cognitive prosthesis. One session contained 4 blocks. Blocks were separated by the presentation of a 58 s fixation point (resting period). One block was composed of 5 matching tasks interleaved with 2 s fixation points. After the presentation of each matching task, the patient was asked to respond as quickly as possible by using her second or third finger to press one of the two buttons on the response pad.

### 2.8. Statistical Analysis

Data analysis was performed using Statistical Package for the Social Sciences (SPSS) for windows version 21 (SPSS Inc., Chicago, IL, USA). Subjects’ data with and without cognitive prosthesis device use were compared with paired T-tests. Healthy subject data are presented as mean ± standard error. *p* values were considered significant if ≤0.05.

## 3. Results

### 3.1. Subject Profiles

The mean age of the healthy subjects (*n* = 6) was 32 ± 3.41 years, and all healthy subjects were women. The patient was a 29-year-old left-handed woman with difficulty memorizing and recalling people’s faces after recovering from a heat stroke at age 16. During the hospitalization, she had drowsiness, with a Glasgow coma scale of E3M6V5 that lasted for 3 days. Her serum creatinine kinase level was 12,975 U/L. The brain MRI showed numerous T2 hyperintense lesions with sizes ranging from 3–16 mm at bilateral cerebral hemispheres, left frontal white matter, thalami, internal capsules, external capsules, midbrain, pons, and cerebellar hemispheres. No surgery was performed at that time. She did not receive any cognitive or motor rehabilitation. Her subsequent facial memory impairment caused her significant inconvenience. She experienced significant social problems because she has difficulty recalling her acquaintances that she needed to interact with in daily life, including colleagues and classmates. She did not have any residual motor, sensory, or coordination deficits.

The patient’s Wechsler Adult Intelligence Scale-IV estimated full-scale IQ was 103, with a Mini-Mental State Examination (MMSE) score of 27. She performed adequately in the judgment of line orientation (27/30, 58 percentile), 3D block construction models (29/29, 53 percentile), and visual form discrimination test (32/32, 53 percentile). However, the patient’s performance in Benton’s facial recognition test was below average (44/54, 25 percentile).

The patient also had below-average performance on executive function (Modified Wisconsin Card Sorting Test complete categories: 6, perseverative errors: 0, non-perseverative errors: 7, and unique errors: 0) and memory tests such as logical memory, visual reproduction, and face-immediate recognition (Wechsler Memory Scale-III logical memory-I 40/75, logical memory-II 18/50, logical memory-recognition 26/30, visual reproduction-I 88/104, visual reproduction-II 40/104, visual reproduction-recognition 45/48, face-immediate recognition 34/48, and face-delay recognition 40/48).

### 3.2. Device Efficacy Assessment

The test results of the healthy subjects are shown in Table 1. It could be observed that device use results in lowered accuracy (attempt 1, *p* = 0.06; attempt 2, *p* < 0.05) and prolonged reaction time in healthy subjects (attempt 1, *p* < 0.01; attempt 2, *p* < 0.05).

Baseline familiar face recognition test results for the patient were obtained during the initial visit. In the first attempt without the device use, the patient’s accuracy was 74.29%, while the reaction time was 6.65 s. In the second attempt, the accuracy was 68.57%, and the reaction time was 8.62 s.

With device use, the patient’s accuracy improved to 94.29%, and reaction time improved to 3.28 s in the first attempt. In the second attempt, the accuracy was 97.14%, and the reaction time was 2.88 s.

Follow-up assessment results for the patient are shown in Table 2. We can observe an improved performance in acquaintance face recognition, both in accuracy and reaction time, over 4 weeks of device use.

#### 3.2.1. Taiwan Face Memory Test

The TFMT [23], a test similar to the Cambridge Face Memory Test [38], was also performed on the patient. The test is essentially a test for unfamiliar face recognition and yields the following results. Healthy subjects’ data obtained from younger adults by Cheng et al. [23] are also shown:

- Stage 1: 72% (13/18) (healthy subjects: 94 ± 5%).
- Stage 2: 50% (15/30) (healthy subjects: 72 ± 16%).
- Stage 3: 42% (10/24) (healthy subjects: 71 ± 15%).
- Total: 52.78% (healthy subjects: 77 ± 12%).

#### 3.2.2. Quality of Life and Other Neuropsychological Assessments

SF-36 was performed and compared against the expected score [39]. The patient had an initial SF-36 physical component summary (PCS) of 50.47 (range of expected score: 20–58) and mental component summary (MCS) of 48.54 (range of expected score: 17–62), suggesting that she had an averagely good quality of life [39]. Changes in quality of life and selected neuropsychological data before and after device use are also shown in Table 2.

### 3.3. Safety and Usability Assessment

No adverse events were reported over the course of the study. The results of the task analysis are shown in Supplementary Table S1 [35]. Usability evaluation revealed that the user experienced slight difficulty during the device operation. The analysis showed that users had no difficulty performing face recognition with the device and obtaining the result using both the device screen and speaker, thanks to the intuitive user interface. The calculated system usability scale (SUS) score was 67.5 [36], suggesting that the device’s usability is generally acceptable. In the post-device use interview, the patient stated that social awkwardness caused by the need to point the hand-held device toward recognition targets for facial recognition negatively affected her user experience.

### 3.4. Machine Learning Performance Assessment

**Figure 3:**
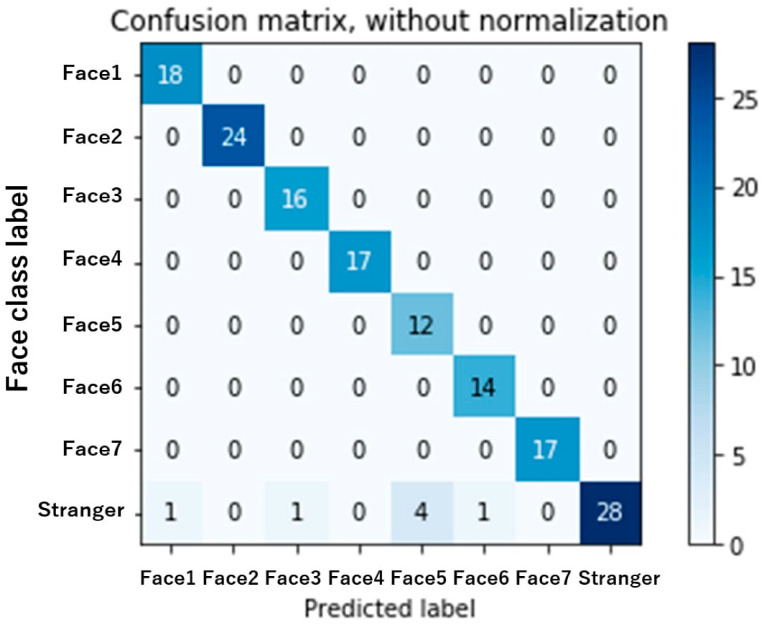
Confusion matrix for the multiclass classification face recognition model algorithm.

The face recognition machine learning algorithm’s confusion matrix during the acquaintance face recognition test, as operated by the patient, is shown in Figure 3.

Real-world device usage data were collected each week throughout the study. Calculated machine learning performance metrics based on the algorithm’s real-world performance are shown in Supplementary Table S2. The real-world cumulative precision of the face recognition machine learning model over 4 weeks was 0.96 ± 0.04, while the cumulative recall was 0.96 ± 0.04.

The data suggested that the cognitive prosthesis was performing reasonably well in a real-world environment. The machine learning performance is achieved using seven face images of each acquaintance as the training set and one face image of each acquaintance as the validation set.

### 3.5. Functional MRI Study Results

**Figure 4:**
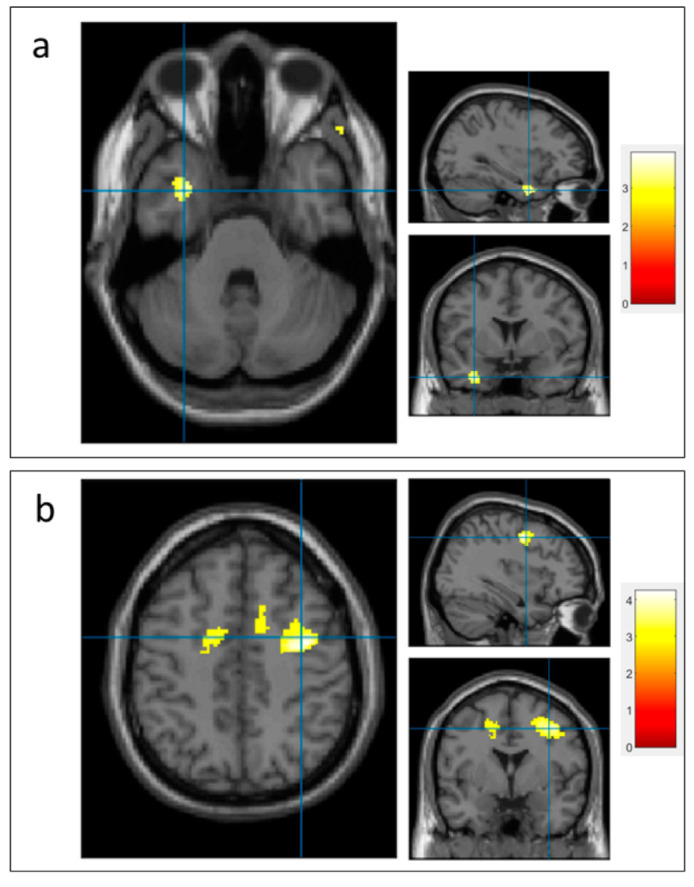
Functional MRI showing brain area activated during the acquaintance face recognition task, without device assistance (**a**) and with device assistance (**b**).

During the acquaintance face recognition task without device assistance, the activated brain area seen on the fMRI was the left superior temporal lobe (Figure 4a). Meanwhile, during the acquaintance face recognition task with device assistance, functional changes were observed in the brain, including the right precentral motor area (Figure 4b).

## 4. Discussion

### 4.1. Face Memory and Recognition in Healthy Subjects

**Figure 5:**
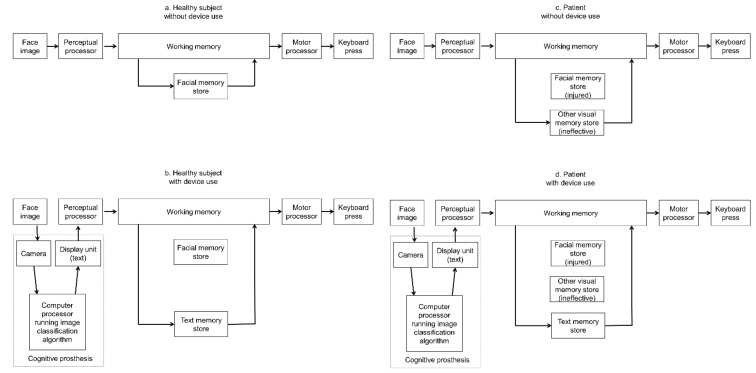
Model human processor schematics for healthy subjects undergoing the acquaintance face recognition test with (**a**) and without (**b**) cognitive prosthesis device use. Schematics for the patient with (**c**) and without (**d**) cognitive prosthesis device use are also shown.

The model human processor [40] schematic for healthy subjects undergoing the acquaintance face recognition test without cognitive prosthesis use is shown in Figure 5a. A previous fMRI study reported that in healthy young subjects the fusiform gyrus was the area activated during face processing [41].

The present study revealed that healthy subjects’ face recognition performance declined with cognitive prosthesis use, pointing out that cognitive prostheses could lower healthy subjects’ cognitive performance accuracy and speed. It could be that when a healthy subject used the cognitive prothesis, the device required additional processing time for face image capture by the camera, running an image processing algorithm by the processor, and text display, as shown in Figure 5b. Although the device could help a healthy subject bypass the cognitive process requiring access to long-term memory, the time saved could not compensate for the time delay caused by the subjects’ interaction time with the device and device processing time. This could have resulted in the longer reaction time by healthy subjects when using the cognitive prosthesis. Kuo et al. have reported that orchestration of the middle frontal cortex (including precentral gyrus), superior temporal cortex, superior parietal cortex, basal temporal area, and extrastriate cortices of the left hemisphere was seen in the fMRI of healthy subjects during reading in Chinese [42].

### 4.2. Efficacy Criteria

#### 4.2.1. Acquaintance Face Recognition Test and Taiwan Face Memory Test

The present study revealed that healthy subjects’ face recognition performance declined following cognitive prosthesis use, pointing out that cognitive prostheses could lower healthy subjects’ cognitive performance accuracy and speed if the cognitive prothesis’ performance happens to be below that of healthy subjects. Nevertheless, the patient’s baseline poor cognitive performance in acquaintance face recognition was ameliorated by device use. The recognition accuracy and reaction significantly improved as measured by the acquaintance face recognition test. The underlying mechanism behind the improved cognitive performance is discussed below together with the fMRI study result. Nevertheless, the patient data indicated that the cognitive prosthesis met both the must-have and nice-to-have need criteria for efficacy for the present study.

On our follow-up evaluation after one month of device use, the repeated acquaintance face recognition test showed an improvement in accuracy and reaction time measured while the patient was not using the device. Nevertheless, the fact that TFMT showed no improvement after 4 weeks suggested that the improved face recognition performance might be for specific acquaintances.

There could be multiple explanations for this observed phenomenon, including training effect and improved test-taking strategies. We have configured the acquaintance test recognition test for the face images to appear in random order and spaced the test for at least one week between the tests to minimize the training effect. However, even though previous studies have questioned the long-term impact of cognitive training, it is also possible that focused cognitive training could lead to improved cognitive performance in a certain domain. More data would be necessary to ascertain the long-term effect of focused cognitive training on patients with cognitive impairment in specific domains.

#### 4.2.2. Quality of Life and Other Neuropsychological Assessments

The patient’s SF-36 MCS component showed no obvious improvement after cognitive prosthesis use, indicating that the cognitive prosthesis device has yet to impact the patient’s life positively. Follow-up for a longer term of device usage is necessary to confirm the validity of this finding. The patient’s modest improvement in MMSE and Benton’s facial recognition test would also need to be replicated in future studies in order to ascertain the cognitive prosthesis’ potential long-term cognitive benefit.

### 4.3. Safety Criteria

The device prototype has met both the must-have and nice-to-have need criteria for safety, as no adverse events are reported during usage.

### 4.4. Usability Criteria

The SUS showed that the system has acceptable usability, indicating that more efforts need to be made to achieve better acceptance. It met the must-have criteria for usability but did not meet the nice-to-have criteria.

The task analysis identified certain factors that affect the user experience in operating the device. In the future, as we expand more functionalities in the device, we would add more tasks into the task analysis section. As the patient revealed during the interview that the need to point the hand-held device toward recognition targets negatively affects the user experience, steps would be taken to address the issue.

### 4.5. Machine Learning Performance Criteria

Analysis of the device’s usage data on the patient’s initial visit and follow-up visits showed that the face recognition machine learning algorithm performed relatively well. The reasonably good performance represents our engineering team’s meticulous effort in building and fine-tuning the machine learning algorithm. The machine learning performance could be the reason behind the device’s positive impact on the patient’s facial recognition performance. It certainly also contributed to the patient’s acceptable response in the usability evaluations. Nevertheless, the algorithm met the must-have criteria for machine learning performance but did not meet the nice-to-have criteria.

### 4.6. Mechanism of Action of Cognitive Prosthesis Based on Functional Study

The brain fMRI study was performed on the patient to study the mechanism that underlies the observed improvement. Analysis of the brain fMRI results reveals the patient’s brain area activated during the matching of names with acquaintances’ face images is the left superior temporal lobe. As mentioned above, a previous fMRI study reported activation of the fusiform face area during the face recognition task in healthy subjects [41]. One explanation for the activation of the left temporal area in our patient might be that the fusiform face area had been damaged due to a previous brain injury. Thus, the patient attempted to access the left temporal lobe to obtain face–name information from the brain area storing long-term memory. Nevertheless, the patient’s poor face recognition performance suggests that the compensation did not work well.

In contrast, the brain area activated during the matching task for the name with the cognitive prosthesis’ recognition result is the precentral gyrus. This is the area shown to be activated during reading Chinese characters in a previous study [42]. Thus, the result supports the hypothesis that the cognitive prosthesis device improves the patient’s facial recognition performance by allowing the patient to skip the reliance on the brain area that functions suboptimally for a certain cognitive task (i.e., using the left temporal lobe for face recognition) and employ the brain area with a preserved function instead (i.e., precentral gyrus).

The model human processor schematic for the patient while not using cognitive prosthesis is shown in Figure 5c; the patient had an injured facial memory store, necessitating her to use the ineffective other visual memory store, causing her to have prolonged facial recognition time and lower recognition accuracy. The device could have allowed the patient to bypass her injured facial memory store and ineffective visual memory store while working on the acquaintance face recognition test, which has resulted in improved facial recognition accuracy and decreased reaction time, as shown in Figure 5d.

### 4.7. Planned Changes to the Prototype

The present study revealed that the device meets both nice-to-have and must-have criteria for efficacy and safety while meeting only must-have criteria for usability and machine learning performance. To meet the nice-to-have criteria for usability, we could improve the design of the device. In particular, changing the design of the device to a wearable form might improve the usability of the device while also decreasing the social awkwardness associated with the device.

Algorithm adjustment could also help meet the nice-to-have criteria for machine learning performance. To further improve the device’s recognition performance, steps that could be taken include upgrading the device’s processing power, adding a proprietary machine learning model training technique to augment the current model, and further optimizing training parameters.

### 4.8. Limitations and Future Directions

The study is an initial effort to explore the potential benefits of cognitive prosthesis in relatively few healthy subjects and a patient. The main limitation of the study is that the patient data are available from a single patient. The difficulty of recruiting patients with face memory impairment suitable for the study, and the longitudinal nature of the study, has prevented the enrollment of more patients. Nevertheless, the valuable data from the single patient could still provide insights into how cognitive prostheses alleviate deficiencies of a domain-specific cognitive impairment.

The work would need to be replicated in a larger number of healthy subjects and patients of both sexes, with different ages and backgrounds, before the cognitive device could be used as a medical device for cognitive assistance and/or rehabilitation. The fact that healthy participants have decreased performance while using the device points out that there is room for further improvement in the device design.

## 5. Conclusions

In sum, the present study has shown that cognitive prosthesis use could improve facial recognition accuracy and reaction time in a patient. The fMRI study result suggests the prosthesis could help the patient bypass the brain area inefficient for facial recognition and employ the area more efficiently for the cognitive task. Follow-up studies revealed improvement of the patient’s facial recognition performance over time. The prosthesis device’s benefit could be supported by real-world device usage data, suggesting a good machine learning performance and usability data showing acceptable usability of the device. The present study is a preliminary study meant to explore the device’s potential benefits and mechanism of action in a small number of young healthy subjects and a patient; more work is needed to confirm the true benefits and more detailed mechanism of action of the cognitive prosthesis. Although the patient data are available from a single patient, they could still provide insights into the potential utilities and mechanisms of action of a domain-specific cognitive prosthesis.

## Figures and Tables

**Table 1:** Healthy subjects’ data for the acquaintance face recognition test (*n* = 6).

	Without Device Assistance		With Device Assistance	
	Accuracy (%)	Reaction Time (s)	Accuracy (%)	Reaction Time (s)
Attempt 1	92.38 ± 4.41	1.27 ± 0.12	80.48 ± 6.23	2.11 ± 0.20 **
Attempt 2	99.05 ± 0.60	1.14 ± 0.11	81.43 ± 5.94 *	2.00 ± 0.21 *

* *p* < 0.05 vs. without device assistance and ** *p* < 0.01 vs. without device assistance.

**Table 2:** Quality of life and selected neuropsychological assessments follow-up data in the patient.

	Baseline	Week 1	Week 2	Week 3	Week 4
**Acquaintance face recognition test (while not using the device)**					
Attempt 1					
Accuracy	74.29%	100%	85.71%	97.14%	100%
Reaction time	6.65	3.54	4.50	1.29	1.23
Attempt 2					
Accuracy	68.57%	100%	100%	100%	100%
Reaction time	8.62	2.19	3.53	1.82	1.55
**Other quality of life and neuropsychological assessments**					
SF-36 PCS	50.47	-	-	-	33.86
SF-36 MCS	48.54	-	-	-	49.76
MMSE	27	-	-	-	30
TFMT	-Stage 1: 13 -Stage 2: 15 -Stage 3: 24 -Total: 52.78%	-	-	-	-Stage 1: 16 -Stage 2: 14 -Stage 3: 7 -Total: 51.39%
Benton’s facial recognition test	44	-	-	-	45

## Data Availability

The data presented in this study are available on request from the corresponding author.

## Supplementary Materials

The following supporting information can be downloaded at: https://www.mdpi.com/article/10.3390/diagnostics12092242/s1, Supplementary Table S1: Task analysis of cognitive prosthesis device; Supplementary Table S2: Real-world cognitive prosthesis algorithm machine learning performance data.
