# Supplementary material for: Domain-Specific Cognitive Prosthesis for Face Memory and Recognition
Source: Diagnostics (Basel). 2022 Sep 16;12(9):2242. doi: 10.3390/diagnostics12092242 (PMC9497523; doi:10.3390/diagnostics12092242)
Supplement: Supplementary file 1 [file diagnostics-12-02242-s001.zip › diagnostics-1852639-supplementary.pdf]

Supplementary Table S1. Task analysis of cognitive prosthesis device.

**Task**	Use error	Total Time	Task analysis
1. Turn on the screen and return to application	0	8.75	Taking 2 attempts to slide up to exit the lock screen
2. Turn off the screen	0	2.19	
3. Face recognition using screen	0	18.05	Taking 16 attempts to slide up to exit the lock screen
4. Face recognition using speaker	0	7.33	
5. Adjusting device volume	0	14.09	
6. Adjusting font size	0	43.07	User randomly tap and slide on screen to find a way to enter the setting screen
7. Recharging the device	0	6.72	

Supplementary Table S2. Real-world cognitive prosthesis algorithm machine learning performance data.

**Metrics**	**Baseline**	**Week 1**	**Week 2**	**Week 3**	**Week 4**	**Cumulative**
Accuracy	0.989	0.994	0.996	0.990	0.967	0.986±0.013
Error rate	0.011	0.006	0.004	0.010	0.033	0.014±0.013
Specificity	0.994	0.997	0.998	0.979	0.980	0.988±0.010
Precision	0.946	0.924	0.982	0.993	0.933	0.958±0.035
Recall	0.975	0.995	0.993	0.933	0.933	0.964±0.035
F1-rate	0.957	0.953	0.987	0.955	0.920	0.954±0.028
False positive rate	0.006	0.003	0.002	0.021	0.020	0.012±0.010
False negative rate	0.025	0.005	0.007	0.067	0.067	0.036±0.035
